# Co-Addition of Mg_2_Si and Graphene for Synergistically Improving the Hydrogen Storage Properties of Mg−Li Alloy

**DOI:** 10.3389/fchem.2021.775537

**Published:** 2021-10-13

**Authors:** Xiantun Huang, Haizhen Liu, Xingqing Duan, Zhiqiang Lan, Jin Guo

**Affiliations:** ^1^ Department of Materials Science and Engineering, Baise University, Baise, China; ^2^ Guangxi Novel Battery Materials Research Center of Engineering Technology, Guangxi Key Laboratory of Processing for Non-Ferrous Metallic and Featured Materials, Guangxi Colleges and Universities Key Laboratory of Novel Energy Materials and Related Technology, School of Physical Science and Technology, Guangxi University, Nanning, China

**Keywords:** hydrogen storage, Mg-Li alloy, doping, graphene, Mg_2_Si

## Abstract

Mg−Li alloy possesses a high hydrogen capacity. However, the hydrogenation and dehydrogenation performances are still far from practical application. In this work, Mg_2_Si (MS) and graphene (G) were employed together to synergistically improve the hydrogen storage properties of Mg−Li alloy. The structures of the samples were studied by XRD and SEM methods. The hydrogen storage performances of the samples were studied by nonisothermal and isothermal hydrogenation and dehydrogenation, thermal analysis, respectively. It is shown that the onset dehydrogenation temperature of Mg−Li alloy was synergistically reduced from 360°C to 310°C after co-addition of Mg_2_Si and graphene. At a constant temperature of 325°C, the Mg−Li−MS−G composite can release 2.7 wt.% of hydrogen within 2 h, while only 0.2 wt.% of hydrogen is released for the undoped Mg−Li alloy. The hydrogenation activation energy of the Mg−Li−MS−G composite was calculated to be 86.5 kJ mol^−1^. Microstructure and hydrogen storage properties studies show that graphene can act as a grinding aid during the ball milling process, which leads to a smaller particle size for the composites. This work demonstrates that coaddition of Mg_2_Si and graphene can synergistically improve the hydrogen storage properties of Mg−Si alloy and offers an insight into the role of graphene in the Mg−Li−MS−G composite.

## 1 Introduction

Hydrogen storage remains a big challenge for the large-scale application of hydrogen energy. Solid-sate hydrogen storage based on the reversible dehydrogenation and rehydrogenation reactions of some materials exhibits promising application potential due to its large volumetric density and sound security. Research and development of advanced hydrogen storage materials is of vital importance for the practical application of solid-state hydrogen storage. During the past decades, many light-weight hydrogen storage materials have been discovered and developed (Li et al., 2019a), for example, light binary metal hydrides (Liu et al., 2020a; Liu et al., 2021a; Zhang et al., 2021a; Huang et al., 2021; Jiang et al., 2021; Si et al., 2021), metal aluminum hydrides (Huang et al., 2020; Li et al., 2020; Ren et al., 2021), metal borohydrides (Zhang et al., 2021b; Neves et al., 2021; Wang and Aguey-Zinsou, 2021; Xian et al., 2021), imides and amides (Chao et al., 2020; Chen et al., 2020; Liu et al., 2021b), etc.

As a potential hydrogen storage material, magnesium hydride (MgH_2_) has attracted considerable interest due to its high gravimetric hydrogen capacity of 7.6 wt.% and the widely availability of Mg. However, the unfavorably high thermal stability and sluggish dehydrogenation kinetics significantly limit its practical application (Luo et al., 2019; Zhang et al., 2020a). Many attempts have been carried out to improve the dehydrogenation and rehydrogeantion properties of MgH_2_, including alloying (Hardian et al., 2018; Hui et al., 2020; Ali and Ismail, 2021), nanoengineering (Zhang et al., 2020a; Zhang et al., 2021c; Thi Thu et al., 2021), and catalyst addition (Zhong et al., 2018; Li et al., 2019b; Liu et al., 2020b; Zhang et al., 2020b; Ding et al., 2020; Singh et al., 2020; Sun et al., 2020; Wang and Deng, 2020; Yao et al., 2020; Zeng et al., 2020; Zhu et al., 2020; Lu et al., 2021a; Lu et al., 2021b; Liu et al., 2021c; Liu et al., 2021), etc. Li is also a H-absorbing metal and has a hydrogen capacity of 11.5 wt.%. However, the dehydrogenation of LiH requires a very high temperature. Mg−Li alloys were reported to possesses better hydrogen storage performances than metals Mg or Li (Lan et al., 2016; Halim and Ismail, 2017; Wu et al., 2018). However, the dehydrogenation properties of Mg−Li alloys are still very poor for practical applications.

To improve the hydrogen storage performances of MgH_2_ and LiH, Vajo et al. (Vajo et al., 2004) utilized Si to destabilize MgH_2_ and LiH. It was shown that the reversibility of the LiH−Si system was improved. The formation of the Mg or Li silicides contributes to the destabilization of MgH_2_ and LiH. Mg_2_Si plays an important role in the destabilized MgH_2_−Si composites. Addition of carbon materials had been considered an effective way to improve the hydrogen storage properties of MgH_2_ or Mg-based hydrogen storage alloys. Lukashev et al. (Lukashev et al., 2006) prepared a Mg−C composite and demonstrated that MgH_2_ was destabilized and the hydrogen dehydrogenation and rehydrogenation kinetics were improved. Another work by Zhang et al. (2015) showed that addition of 10 wt.% graphene significantly enhances the dehydrogenation thermodynamics and kinetics of MgH_2_. Similar results were also reported by Imamura et al. (1999). Besides graphene, carbon black, graphite, and carbon nanotubes all have positive influence on the hydrogen storage performance of MgH_2_ (Huang et al., 2007). It was suggested that the interaction between MgH_2_ and carbon materials contributes to the improvement of the hydrogen storage performances of MgH_2_.

Inspired by the above-mentioned discussions, we performed a systematic investigation on the effects of Mg_2_Si and graphene on the hydrogenation and dehydrogenation properties of Mg−Li alloys in this work. The following will show that Mg_2_Si and graphene have synergetic enhancing effect on the dehydrogenation properties of MgH_2_. Such fundamental investigation may provide useful guidance for the design and development of high-performance Mg−Li alloys.

## 2 Experimental Details

### 2.1 Materials Synthesis

To prepare the Mg−Li alloy (Mg_77_Li_23_), the powders of Mg (99.5% of purity from Alfa Aesar) and LiH (99.7% of purity from Alfa Aesar) with a molar ratio of Mg_77_Li_23_ were first homogeneously mixed and then pressed into plates using a pressure of 20 MPa. The plates were subject to sintering treatment at 500°C for 2 h with a high-vacuum furnace. After that, the plates were mechanically crushed and then mixed with Mg_2_Si (MS), graphene (G), and Mg_2_Si + graphene (MS + G), respectively, with a content of 5 wt.%. The mixtures were then ball-milled to prepare various composites, that is Mg_77_Li_23_ + 5 wt.% Mg_2_Si (denoted as Mg−Li−MS), Mg_77_Li_23_ + 5 wt.% graphene (denoted as Mg−Li−G), and Mg_77_Li_23_ + 5 wt.% (Mg_2_Si + graphene) (denoted as Mg−Li−MS−G). For comparison, the as-prepared Mg_77_Li_23_ alloy was also ball-milled under the same conditions, which denoted as Mg−Li. The ball-to-powder weight ratio was about 40:1, and the milling process was carried out at 300 rpm for 30 h. The preparation of all the samples and their handling were carried out without exposure to air.

### 2.2 Materials Characterizations

The dehydrogenation/hydrogenation were performed on a Sieverts-type apparatus by a volumetric method. During measurements, the temperature and pressure data were collected by a computer. The amounts of hydrogen desorbed and absorbed were calculated by the ideal gas equation using the obtained temperature and pressure data. The hydrogenation measurements were conducted using an initial hydrogen pressure of 6.0 MPa, and the samples were heated gradually from room temperature to 377°C with a heating rate of 2°C min^−1^. The isothermal dehydrogenation measurements were started at an initial hydrogen pressure of 1.1 kPa. The nonisothermal dehydrogenation measurements were conducted as the isothermal measurements, with the temperature increased from room temperature to a target temperature using a heating rate of 2°C min^−1^. To further determin the dehydrogenation behavior of the composites, the fully hydrogenated samples were measured by using a differential scanning calorimeter (Linseis STA PT-1000) with a high-purity hydrogen (99.999%) of 30 mL min^−1^ as the carrying gas. Powder X-ray diffraction (XRD) measurements were carried out using a Rinku Miniflex-600 diffractometer with Cu-Kα radiation. To avoid exposure to any moisture or oxygen, the samples were sealed with an amorphous membrane in an Ar glovebox prior to measurements. The morphologies of the samples were observed by a scanning electron microscopy (JEOL, JSM-6510).

## 3 Results and Discussion

**FIGURE 1:**
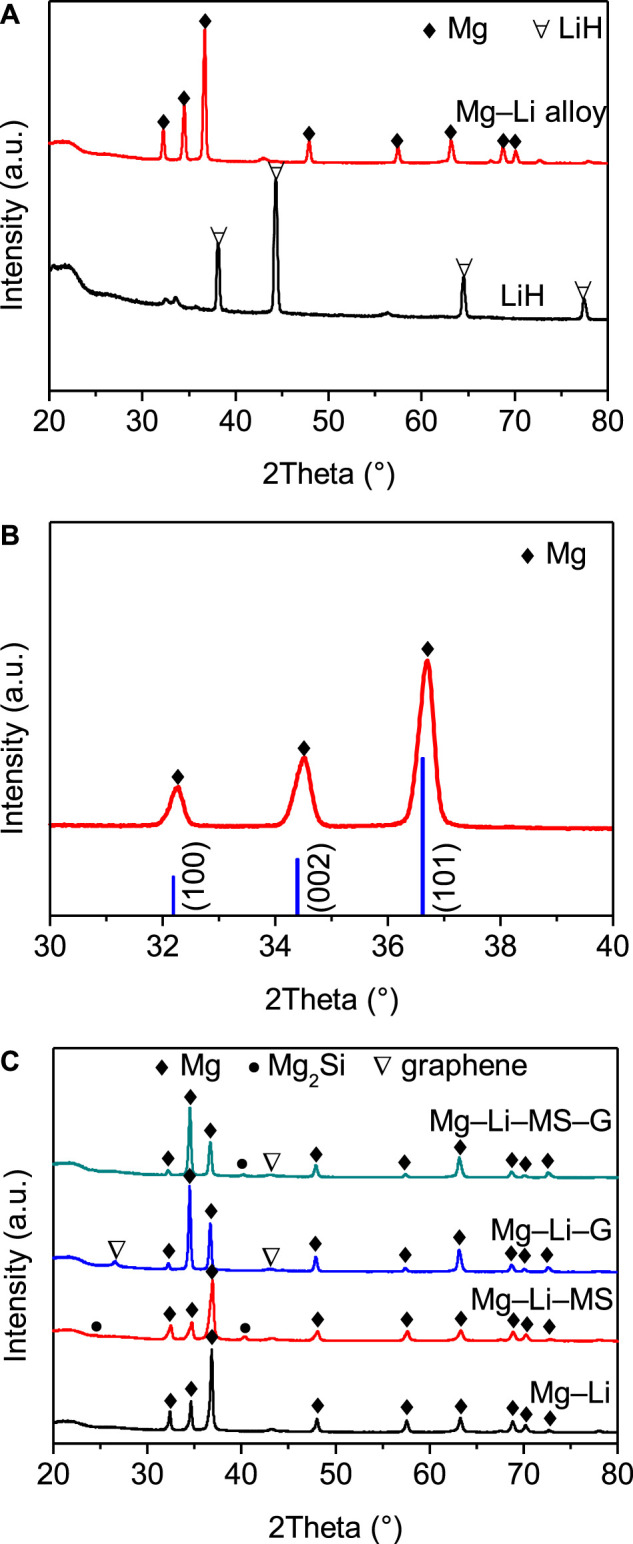
**(A)** XRD patterns of as-received LiH and Mg−Li alloy after sintering treatment; **(B)** Expanded XRD patterns of Mg−Li alloy after sintering treatment in 2Theta rage 30–40°; **(C)** XRD patterns of as-milled Mg−Li, Mg−Li−MS, Mg−Li−G, and Mg−Li−MS−G composites.

Figure 1A shows the XRD pattern of Mg−Li alloy after sintering treatment and the as-received LiH for comparison. It can be seen that only Mg peak is detected, and the peak of LiH disappears in the sintered Mg−Li alloy. Figure 1B displays the expanded XRD pattern of the Mg−Li alloy. The standard XRD peak position of Mg is also displayed for reference. It is shown that the peaks of Mg in the Mg−Li alloy all shift to higher angles, which indicates a shrinkage of the Mg crystal structure according to the Bragg’s equation. This suggests that the Li may has totally dissolved into the Mg lattice to form a solid solution phase of Mg since the atomic radius of Mg is larger than Li. Figure 1C shows the XRD patterns of the as-milled Mg−Li, Mg−Li−MS, Mg−Li−G, and Mg−Li−MS−G composites. It can be seen that Mg phase is detected in all samples. Besides, some minor peaks of the additives (Mg_2_Si and graphene) remain nearly unchanged in the corresponding milled composites. This means that Mg_2_Si and graphene do not react with the Mg−Li alloy.

**FIGURE 2:**
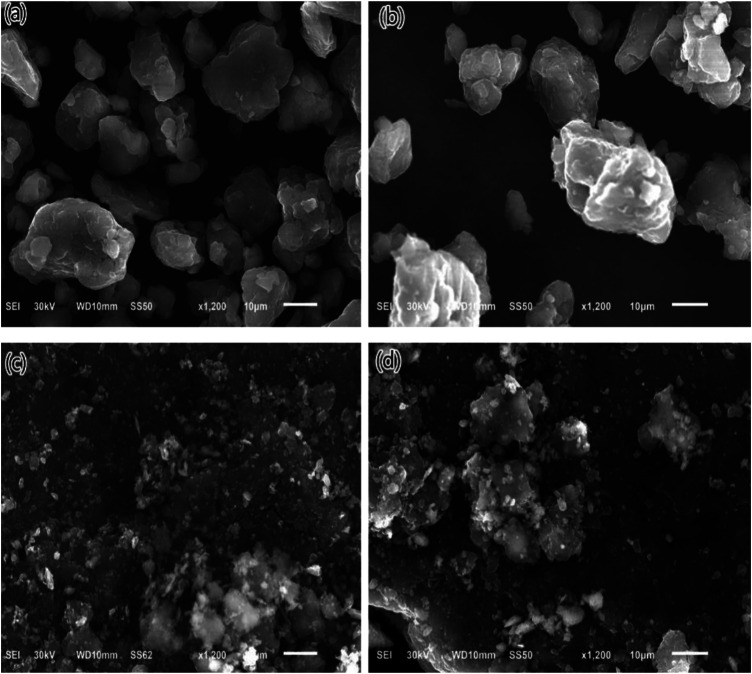
SEM images of as-milled Mg−Li **(A)**, Mg−Li−MS **(B)**, Mg−Li−G **(C)**, and Mg−Li−MS−G **(D)** composites.

Figure 2 presents the SEM images of the as-milled Mg−Li, Mg−Li−MS, Mg−Li−G, and Mg−Li−MS−G composites. For the samples without graphene addition, as shown in Figures 2A,B, large particles and the severe agglomeration of small particles can be observed. These large particles are formed from the agglomeration of smaller ones. For the samples with graphene addition, as shown in Figures 2C,D, it is obvious that the particle size is much smaller than the previous two samples, which further indicates the graphene can act as an important grinding aid and significantly improve the milling efficiency.

**FIGURE 3:**
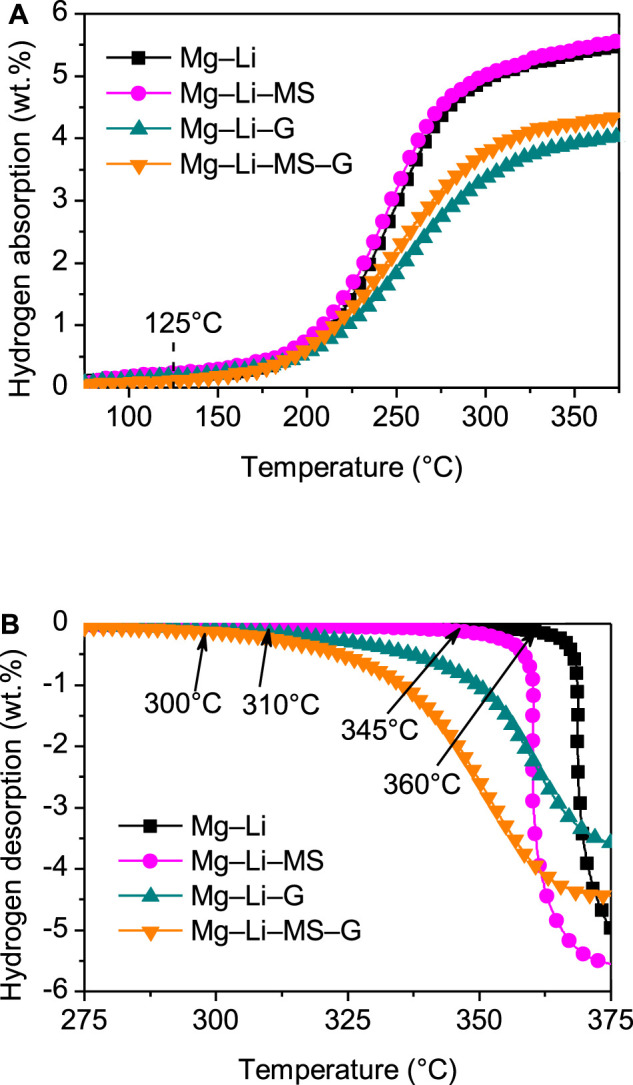
Hydrogenation **(A)** and dehydrogenation **(B)** curves of the Mg−Li, Mg−Li−MS, Mg−Li−G, and Mg−Li−MS−G composites with a heating rate of 2°C min^−1^.

To understand the effects of Mg_2_Si, graphene and Mg_2_Si + graphene on the hydrogenation/dehydrogenation characteristics of the Mg−Li alloys. The hydrogenation and dehydrogenation curves of the various composites are shown in Figures 3A,B, respectively. The hydrogenations of the composites all start at about 125°C, implying that the starting temperature of the hydrogenation is not affected by addition of Mg_2_Si or graphene. However, the hydrogenation capacities for samples with graphene (4.1 wt.%) or Mg_2_Si + graphene (4.4wt.%) addition is slightly lower than the other two samples, which may be due to the fact that the graphene does not absorb any hydrogen under such conditions. The final hydrogenation capacities are 5.9 wt.%, 6.0 wt.%, 4.4 wt.%, and 4.6 wt.% for the Mg−Li, Mg−Li−MS, Mg−Li−G, and Mg−Li−MS−G composites, respectively. Figure 3B displays the dehydrogenation curves of the various composites. The onset dehydrogenation temperatures of the Mg−Li alloys are substantially reduced from 360°C to 300°C, 310°C, and 345°C, respectively, for samples with MS, G or MS + G additions). Obviously, the Mg−Li−G−MS exhibits the lowest onset dehydrogenation temperature among the various composites, which indicates a synergistic improving effect of M_2_Si and graphene on the dehydrogenation of Mg−Li alloy. The significant improvement in kinetics of the graphene-containing samples may be due to the smaller particle size as shown in Figure 2. The smaller particles will result in more fresh surface exposures, which leads to the improved dehydrogenation performances of Mg−Li alloy. The final dehydrogenation capacities are 5.7 wt.%, 5.6 wt.%, 3.9 wt.%, and 4.5 wt.% for the Mg−Li, Mg−Li−MS, Mg−Li−G, and Mg−Li−MS−G composites, respectively.

**FIGURE 4:**
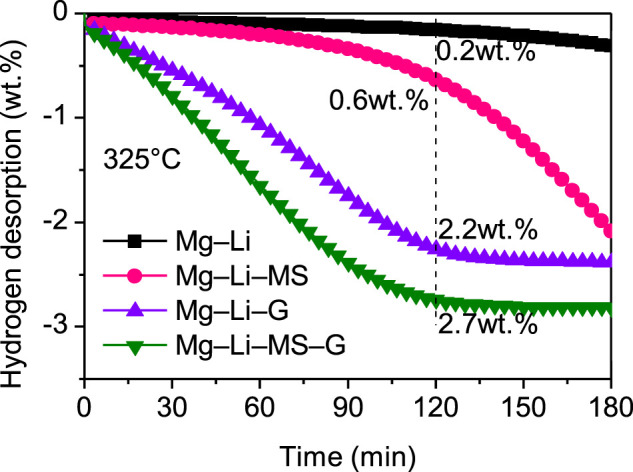
Isothermal dehydrogenation curves of the Mg−Li, Mg−Li−MS, Mg−Li−G, and Mg−Li−MS−G composites at a constant temperature of 325°C.

To investigate the dehydrogenation kinetics of the various composites, isothermal dehydrogenation measurements were carried out. Figure 4 displays the isothermal dihydrogen curves of the various composites at a constant temperature of 325°C. It is found that the dehydrogenation kinetics of the Mg−Li alloy was significantly improved by the addition of Mg_2_Si or graphene. Comparatively, the as-milled Mg−Li alloy without additive can hardly release hydrogen at 325°C. After dehydrogenation for 2 h, the dehydrogenation capacity reaches 0.6 wt.%, 2.2 wt.%, and 2.7 wt.% for the Mg−Li−MS, Mg−Li−G, and Mg−Li−MS−G composites, respectively. Obviously, the composite with co-addition of Mg_2_Si and graphene exhibits the best dehydrogenation kinetics, which also imply a synergistic effect of Mg_2_Si and graphene on the dehydrogenation of the Mg−Li alloy. It should be noted that the onset dehydrogenation temperature of Mg−Li−MS composite is as high as 345°C shown in Figure 3B; this is because the dehydrogenation needs an induction period. When the composite starts dehydrogenation, the temperature has already increased to 345°C. Therefore, at a constant temperature of 325°C, the composite still can release hydrogen as long as enough reaction time was provided.

**FIGURE 5:**
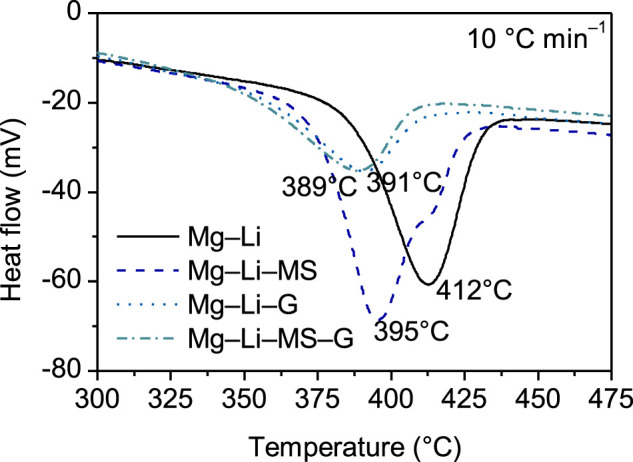
DSC curves of the Mg−Li, Mg−Li−MS, Mg−Li−G, and Mg−Li−MS−G composites with a heating rate of 10°C min^−1^.

The dehydrogenation performance of the various composites were further studied by thermal analysis. Figure 5 illustrates the DSC curves of the various composites. It is interesting to find that addition of Mg_2_Si or graphene substantially decreases the peak dehydrogenation temperature of the Mg−Li alloy. For the as-milled Mg−Li alloy, the peak dehydrogenation occurs at 412°C, while for the samples with addition of Mg_2_Si, graphene or Mg_2_Si + graphene, their peak dehydrogenation temperatures shift to 395, 391, or 389°C, respectively. The enhance in dehydrogenation kinetics is attributed to the reduction of the particle size. As can be seen in Figure 2, graphene can play a grinding aid role during the mechanical milling, resulting in fine particle size. The smaller particle size can shorten the diffusion path of the hydrogen atom. In other word, the fine particle may reduce the barrier for the diffusion of the hydrogen atoms. As point out in the work by Huang et al. (Huang et al., 2007), the cleavage-decomposed graphite in the composites may intimately interacts with finely divided particles via charge-transfer reactions. Such charge-transfer sites are also responsible for the catalytic activation of hydrogen molecules. Consequently, the dehydrogenation properties of Mg−Li composites were improved.

**FIGURE 6:**
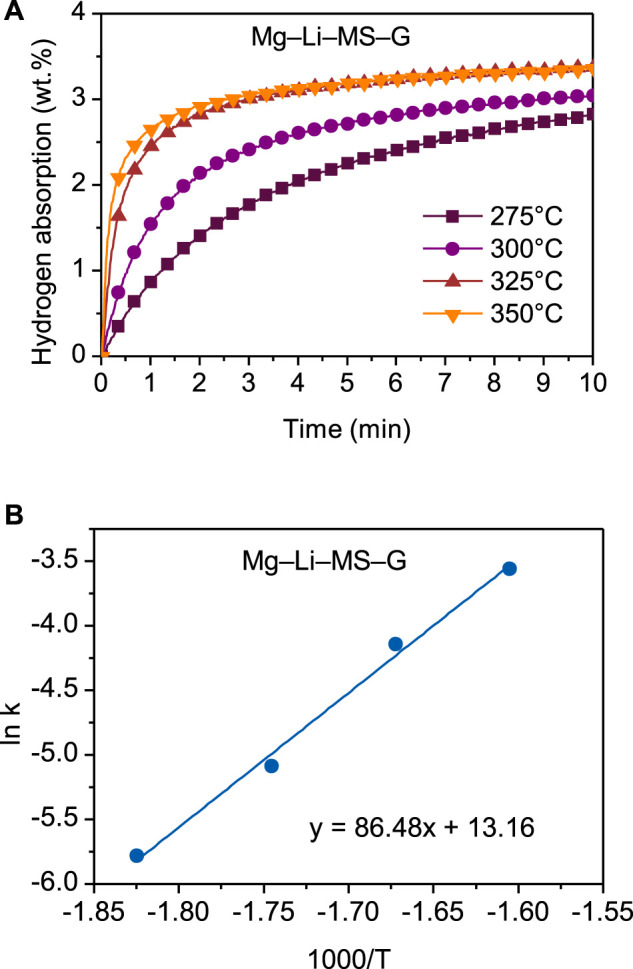
Hydrogenation curves under different temperatures **(A)** and Arrhenius’s plot **(B)** of the Mg−Li−MS−G composite.

The Mg−Li−MS−G composite possesses the best hydrogen storage performances among the various composites. Therefore, the energy barrier was further studied by calculating the activation energy for hydrogenation. Figure 6A shows the hydrogenation curves of the Mg−Li−MS−G composite at different temperatures. A two-step hydrogen absorption process is distinctly observed in the temperature range 275–350°C. In the first step, the hydrogen atoms are quickly absorbed at the particles surface with small increase in temperature. In the second step, the hydrogen atoms start to diffusion in the bulk material, resulting in a lower absorption rate. As the operating temperature increases, the hydrogenation rate is distinctly accelerated. The discrepancy between the first and second steps suggests that the hydrogenation of the Mg−Li−MS−G composite is governed by the diffusion of hydrogen in the bulk material. For the analysis of the apparent activation energy, the Johnson-Mehl-Avrami (JMA) formula is adopted (Avrami, 1939; Kempen et al., 2002). The equation can be written as

ln[−ln(1−α)]=η⁡ln⁡k+η⁡ln⁡t, (1)

Where *α* is the fraction already reacted at time *t*, *η* is the Avrami exponent, and *k* is the rate constant. Based on Eq. 1, the temperature-dependent reaction rate exponent, *k*, can be determined. It should be noted that the data in *α* range of 0.2–0.8 was selected for fitting by JMA equation. It is well known that the temperature-dependent reaction rate exponent, *k*, satisfies the Arrhenius equation, which is expressed as follows:

$$k=A\exp\left(E_{a}RT\right),$$ (2)

in which *A* is the pre-exponential factor, *E*_a_ is the apparent activation energy, *R* is the gas constant, and *T* is the absolute temperature. Figure 6B shows the Arrhenius’s plot for the Mg−Li−MS−G composite. Clearly, the plot of ln*k* versus 1/*T* exhibits a good linearity. The apparent activation energy *E*_*a*_ for the Mg−Li−MS−G composite was calculated to be 86.5 kJ mol^−1^. This is significantly lower than that of Mg/MgH_2_ (95–130 kJ mol^−1^) (Fernandez and Sanchez, 2002; Norberg et al., 2011). The reduction of the activation energy contributes directly to the improvement of the hydrogen storage performances of the Mg−Li−MS−G composite.

## 4 Conclusion

Mg_2_Si and graphene was utilized to improve the hydrogenation and dehydrogenation performances of the Mg−Li alloy. The hydrogenation of the Mg−Li alloy was not affected by the addition of Mg_2_Si or graphene. However, the dehydrogenation of the Mg−Li alloy was synergistically improved by co-addition of Mg_2_Si and graphene. The onset dehydrogenation temperature of Mg−Li alloy was reduced by 50°C after co-addition of Mg_2_Si and graphene. At 325°C, the Mg−Li−MS−G composite can release 2.7 wt.% of hydrogen within 2 h. The hydrogenation reaction activation energy of the Mg−Li−MS−G composite was calculated to be 86.5 kJ mol^−1^. Graphene can act as a grinding aid during the ball milling process, which leads to a smaller particle size of the Mg−Li alloy. Detailed enhancing mechanism inside the Mg−Li−MS−G composite needs to be studied further to understand the exact role of Mg_2_Si and graphene in tailoring the hydrogen storage properties of the Mg−Li alloy.

## Data Availability

The raw data supporting the conclusion of this article will be made available by the authors, without undue reservation.
